# Oxidative Stress, DNA Damage, and c-Abl Signaling: At the Crossroad in Neurodegenerative Diseases?

**DOI:** 10.1155/2012/683097

**Published:** 2012-06-18

**Authors:** Stefania Gonfloni, Emiliano Maiani, Claudia Di Bartolomeo, Marc Diederich, Gianni Cesareni

**Affiliations:** ^1^Department of Biology, University of Rome “Tor Vergata,” Via della Ricerca Scientifica, 00133 Rome, Italy; ^2^Laboratoire de Biologie Moléculaire et Cellulaire du Cancer, Fondation de Recherche Cancer et Sang, Hôpital Kirchberg, 9 Rue Edward Steichen, 2540 Luxembourg, Luxembourg

## Abstract

The c-Abl tyrosine kinase is implicated in diverse cellular activities including growth factor signaling, cell adhesion, oxidative stress, and DNA damage response. Studies in mouse models have shown that the kinases of the c-Abl family play a role in the development of the central nervous system. Recent reports show that aberrant c-Abl activation causes neuroinflammation and neuronal loss in the forebrain of transgenic adult mice. In line with these observations, an increased c-Abl activation is reported in human neurodegenerative pathologies, such as Parkinson's, and Alzheimer's diseases. This suggests that aberrant nonspecific posttranslational modifications induced by c-Abl may contribute to fuel the recurrent phenotypes/features linked to neurodegenerative disorders, such as an impaired mitochondrial function, oxidative stress, and accumulation of protein aggregates. Herein, we review some reports on c-Abl function in neuronal cells and we propose that modulation of different aspects of c-Abl signaling may contribute to mediate the molecular events at the interface between stress signaling, metabolic regulation, and DNA damage. Finally, we propose that this may have an impact in the development of new therapeutic strategies.

## 1. Introduction 

A broad range of pathological disorders is linked to oxidative stress, including carcinogenesis and several age-dependent disorders (i.e., as neurodegenerative diseases). Oxidative stress is defined as an imbalance in which the production of reactive oxygen species (ROS) overcomes the antioxidative cell defence system. Oxidative stress can be induced by exogenous and endogenous sources. For instance, hydrogen peroxide and chemotherapeutic reagents are exogenous sources of ROS, whereas mitochondrial energy metabolism is considered a major source for the production of ROS within the cell [1]. ROS can directly react with macromolecules, such as DNA, lipids, and proteins. Oxidative DNA lesions, if unrepaired, can induce mutations and deletions in both nuclear and mitochondrial genomes [2] and chromosomal abnormalities. Cells are also very sensitive to lipid peroxidation [3] and most amino acid residues in a protein can be oxidized by ROS. Often these modifications impair protein function [4]. Antioxidant defences are built in a complex network of nonenzymatic and enzymatic components of the cell. This network has been extensively reviewed [5, 6]. In short, Glutathione (GSH) is a nonenzymatic antioxidant, which acts in the cellular thiol/disulfide system, with the ratio of GSH to GSSH (glutathione disulphide) mirroring the redox status of the cell. On the other hand, enzymatic antioxidants include superoxide dismutases SODs, catalase, peroxiredoxins (PRxs), and glutathione peroxidases (GPx). The toxicity of ROS is only one facet of their action. ROS are also produced at low level within the cell, where they can play an important role in the redox-dependent regulation of signaling [7]. Hence, ROS are implicated in several cellular processes, including cell proliferation, cell cycle arrest, and programmed cell death [8]. Cellular responses to DNA damage or oxidative stress are critical for survival, and the direct link between ROS and oxidative DNA damage indicates the interplay of ROS signaling with the DNA damage response (DDR) [9]. Evidence indicates the involvement of the phosphatidylinositol-3-kinases- (PI3K-) related kinases, Ataxia telangiectasia mutated (ATM), DNA-dependent protein kinase catalytic subunit (DNA-PKcs), and ATM- and Rad-3 related (ATR) in oxidative DNA lesion repair and signaling response [10]. This finding together with the emerging role of c-Abl in the DDR [11] and in oxidative DNA damage [12] seems to point out a role for these DDR kinases as “sensors” for redox signaling. In particular, herein we discuss how an aberrant (nonspecific) c-Abl signaling may contribute to maintain high levels of ROS that in turn can damage organelles, mitochondria, and DNA, with these effects ending towards neuronal degeneration. 

## 2. ROS and c-Abl Signaling 

Oxidative stress contributes to the pathogenesis of a large number of human disorders. No doubt that a better understanding of the controlled production (and of regulatory targets) of ROS should provide the rationale for novel therapeutic treatments [13]. ROS signaling is reversible, tightly controlled through a regulatory network. This network results from a concerted assembly of protein complexes, built through protein interactions mediated by interaction modules and posttranslational modifications in the binding partners. Protein modularity and the reversible nature of posttranslational modifications allow the dynamic assembly of local temporary signaling circuits regulated by feedback controls. The strength and the duration of redox signaling are regulated *via* the oxidative modifications of the kinases and phosphatases that in turn control the activity of enzymes involved in antioxidant activities and vice versa. Oxidant level modulates c-Abl activity [14, 15]. In turn, c-Abl can interact (and regulate) with several enzymes implicated in controlling the redox state of the cell. One of them, the catalase is an immediate effector of the antioxidant cellular defense by converting H_2_O_2_ to H_2_O and O_2_ in the peroxisomes. c-Abl and the product of the c-Abl-related gene (Arg) target catalase on the two residues Y321 and Y386 leading to its ubiquitination and to a consequent proteasomal-dependent degradation of the enzyme [16]. Similarly, c-Abl-deficient cells display a higher level of expression of the antioxidant protein peroxiredoxin I (Prx1) [17]. Prx1 is considered a physiological inhibitor of c-Abl. Prx1 interacts with the SH3 domain of c-Abl and inhibits its catalytic activity [18]. Depending on the oxidative level in the cell, glutathione peroxidase1 can be phosphorylated on Tyr-96 and activated by c-Abl/Arg [19]. In short, c-Abl activation has mostly a negative effect on enzymes involved in the antioxidant defence, with rare exceptions. Moreover, c-abl, as a component of redox regulatory circuits, can be modified by *S*-glutathionylation, with this reversible modification leading to downregulation of its kinase activity [20]. 

## 3. c-Abl Signaling in Neurodegenerative Disease

Oxidative stress, accumulation of protein aggregates, and damaged mitochondria are common hallmarks of neurological diseases. Aberrant c-Abl activation is linked to many neuronal disorders as recently reviewed by Schlatterer and coworkers [21]. In the brain, c-Abl activation can be monitored by specific antibodies, which target phosphorylated residues present only in the active conformation of the kinase. Staining with these phosphoantibodies indicates that c-Abl colocalized with granulovacuolar degeneration (GVD) in brains of human Alzheimer (AD) patients. Moreover, c-Abl phosphorylated at T735, a site required for binding 14-3-3 in the cytosol [22], colocalized with amyloid plaques, neurofibrillary tangles (NFTs), and GVD in the entorhinal cortex and hippocampus and brain of AD patients [21, 23]. Tau phosphorylation mediated by c-Abl is detected in NFTs in Alzheimer disease [21, 24, 25]. Oxidative stress activates c-Abl in neuronal cells [26] and amyloid *β* results in increased expression of c-Abl and p73 [27]. Amyloid-*β* (A*β*) fibrils in primary neurons induce the c-Abl/p73 proapoptotic signaling, while STI571, a pharmacological c-Abl inhibitor, prevents Amyloid *β*-dependent toxicity [26]. The c-Abl/p73 proapoptotic pathway is also targeted in the cerebellum of Niemann-Pick type C (NPC) mice [28]. Niemann-Pick type C (NPC) is a neurodegenerative disorder characterized by intralysosomal accumulation of cholesterol leading to neuronal loss. Pharmacological inhibition of c-Abl with STI571 rescues Purkinje neurons, reduces general cell apoptosis in the cerebellum, improves neurological symptoms, and increases the survival of NPC mice [29]. Evidence indicates that c-Abl binding with p73 is induced by ROS, with NAC (the oxidant scavenger N-acetylcysteine) treatment reducing the c-Abl/p73 activation as well as the levels of apoptosis in NPC neurons [28].

Recent findings indicate that some effects of c-Abl induced by glucose metabolism might be mediated through p53 phosphorylation. In fact, c-Abl is involved in high glucose-induced apoptosis in embryonic E12.5 cortical neural progenitor cells (NPCs) derived from mice brain. Once more again, inhibition of c-Abl by ST571 reduced apoptosis in NPCs by preventing the nuclear protein accumulation of p53 in response to high glucose [30]. Moreover, administration of reactive oxygen species scavengers impairs the accumulation of c-Abl and p53 leading to a decreased NPCs apoptosis. In human neuroblastoma (SH-SY5Y) cells, c-Abl targets cyclin-dependent kinase 5 (Cdk5) on tyrosine residue Y15 in response to oxidative stress by hydrogen peroxide. In turn, Cdk5 can modulate p53 levels and p53 activity. Hence, both c-Abl and Cdk5 cooperatively mediate p53 transcriptional activation resulting in neuronal death [31]. A recent study also indicates that hyperglycemia-induced apoptosis of NPCs is mediated by a PKC*δ*- (Protein-Kinase C-delta-) dependent mechanism [32]. Tyrosine phosphorylation of PKC*δ* by c-Abl is important for the translocation of the PKC*δ*-Abl complex from the cytoplasm to the nucleus. Downregulation of PKC*δ* (or c-Abl) or inhibition of c-Abl by STI571 can decrease this translocation, impairing p53 accumulation in the nucleus of NPCs [32].

A redox imbalance is apparently a predominant feature of brains of individuals with Parkinson's disease (PD). Evidence derived from postmortem studies indicates an increased oxidation of lipids, proteins and DNA, a severe decrease in GSH concentration, and an accumulation of SOD2 (see [33] and references therein). Oxidative DNA damage occurs to a higher extent in Parkinson's disease individuals compared with age-matched controls [34]. Brains of Parkinson's patients are also enriched in autophagosome-like structures reminiscent of autophagic stress. Interestingly, inherited forms of Parkinson's disease are associated with loss-of-function mutations in genes encoding proteins that target the mitochondria and modulate autophagy, including the E3 ubiquitin ligase parkin (see [33] and references therein). c-Abl phosphorylates parkin on Y143 and inhibits parkin's ubiquitin E3 ligase activity and its protective function. Conversely, STI-571 treatment prevents the phosphorylation of parkin, maintaining it in a catalytically active state. Interestingly, the protective effect of STI-571 is not observed in parkin-deficient cells. Conditional knockout of c-Abl also prevents the phosphorylation of parkin, the accumulation of its substrates, and results in neurotoxicity in response to 1-methyl-4-phenyl-1,2,3,6-tetrahydropyridine (MPTP) intoxication [35]. Briefly, STI-571 prevents tyrosine phosphorylation ofparkinand restores its E3 ligase activity and cytoprotective function both *in vitro* and *in vivo*. Compelling evidence indicates that tyrosine phosphorylation ofparkinby c-Ablis a major posttranslational modification that leads to loss of parkinfunction and disease progression in sporadic PD [36]. Moreover, a selective inhibition of c-Abloffers new therapeutic strategies for blocking PD progression [35, 36]. Another level of c-Abl-dependent-regulation impinges on the activation of PKC*δ*. In cell culture models of PD, oxidative stress activates PKC*δ* through a caspase-3-dependent proteolytic cleavage inducing apoptotic cell death [37, 38]. Interestingly proteolytic activation of PKC*δ* is regulated through phosphorylation of its tyrosine residues [39]. Evidence regarding a functional interaction between PKC*δ* and c-Abl has been provided following oxidative stress response [14]. c-Abl phosphorylates PKC*δ* on tyrosine 311, with this modification contributing to the apoptotic effect of hydrogen peroxide [40]. On the other hand, ST571 can block PKC*δ* activation protecting cells from apoptosis [41]. Moreover, Xiao et al. identified c-Abl as a novel upstream activator of the protein kinase MST1 (mammalian Ste-20 like kinase1) that plays an essential role in oxidative-stress-induced neuronal cell death. Upon phosphorylation of MST1 at Y433 by c-Abl, authors demonstrated activation of FOXO3 that leads eventually to neuronal cell death. The latter mechanism is inhibited either by STI571 or c-Abl knockdown [42].

In short, this combined evidence stresses the physiological relevance of the interface between c-Abl signaling and redox state, metabolic regulation and DNA damage response mediated by transcription factors, such as FOXO-3 or members of the p53 family. 

**Figure 1:**
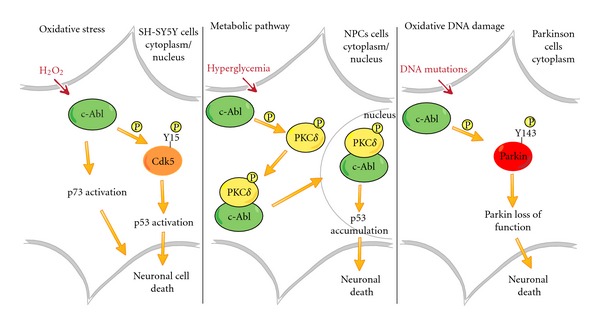
The figure illustrates the involvement of c-Abl in many cellular stress pathways. Oxidative stress, hyperglycemia, and DNA damage response induce c-Abl activation. In human neuroblastoma (SH-SY5Y cells), c-Abl targets p73, promoting neuronal death in response to hydrogen peroxide. In addition, c-Abl can also phophorylate Cdk5 and in tandem with Cdk5 can mediate p53 activation, promoting neuronal death. Hyperglycemia-induced apoptosis of NPCs is mediated by the translocation of the PKC*δ*-Abl complex to the nucleus. This translocation impacts on p53 activation leading to neuronal death. Oxidative DNA damage in Parkinson disorder is associated with increased c-Abl activity. c-Abl mediates tyrosine phosphorylation of Parkin and inhibits parkin's ubiquitin E3 ligase activity.

The dynamic of each signal-transduction path seems to be governed by a small set of recurring c-Abl-mediated regulatory circuits, that depending on their subcellular localization and response duration may result in neuronal death (Figure 1). Of note, inactivation of c-Abl by STI571 can have a protective effect and can reduce neuronal loss. 

## 4. Autophagy, Mitochondria, and Oxidative Stress: Cross Talk with c-Abl Signaling 

Protein aggregation and organelle dysfunction are peculiar hallmarks of many late-onset neurodegenerative disorders. Mitochondrial damage and dysfunction is indeed linked to neurodegeneration in a variety of animal models [33]. Clearance of misfolded proteins and damaged organelles may be considered an effective recovery strategy for stressed neuronal cells [43]. Autophagy is a lysosome-dependent pathway involved in the turnover of proteins and intracellular organelles. It is becoming increasingly evident that induction of a certain level of autophagy may exert a neuroprotective function, while its inappropriate or defective activation may result in neuronal cell loss in most neurodegenerative diseases [44]. Abnormal autophagosomes are frequently observed in selective neuronal populations afflicted in common neurodegenerative diseases, such as Alzheimer's disease, Parkinson's disease, Huntington's disease, and amyotrophic lateral sclerosis. However, whether accumulation of autophagosomes plays a protective role or rather contributes to neuronal cell death is still a controversial issue [44, 45]. Despite this uncertainty, an accurate titration of autophagy should favor a neuroprotective response. In particular, if it is strictly modulated through an efficient concerted action of the complex autophagy machinery. ROS can induce autophagy [46]. In addition, inhibition, depletion, or knockout of the c-Abl family kinases, c-Abl and Arg, resulted in a dramatic reduction in the intracellular activities of the lysosomal glycosidases alpha-galactosidase, alpha-mannosidase, and neuraminidase. Inhibition of c-Abl kinases also reduced the processing of the precursor forms of cathepsin D and cathepsin L to their mature, lysosomal forms, leading to an impaired turnover of long-lived cytosolic proteins and accumulation of autophagosomes [46, 47]. Together all these findings suggest a positive role for c-Abl kinases in the regulation of autophagy with important implications for therapies [46]. 

In conclusion, many observations indicate that c-Abl activity is increased in human neurodegenerative diseases (Alzheimer, Parkinson, and tauopathies). However, where c-Abl meets the cascade of events underlying neurodegenerative disorders remains still elusive. A plausible scenario implies the involvement of c-Abl on multiple interconnected pathways eventually acting as an arbiter of neuronal survival and death decisions, most likely playing with autophagy, metabolic regulation and DNA damage signaling response. In adult mouse models, aberrant c-Abl activation causes neurodegeneration and neuroinflammation in forebrain neurons, thus implying c-Abl as a possible target for therapeutic treatments [21]. Several reports have shown that c-Abl plays distinct roles based upon its subcellular localization. Is the achievement of a certain/specific relocalization of c-Abl required for the development of the neuronal disease? The interplay between cytoplasmic, nuclear and mitochondrial localization of c-Abl is an important aspect for oxidative stress-induced apoptosis. In concert with this, c-Abl catalytic outcomes are strictly associated with its subcellular localization. TTK, also known as PYT (Phosphotyrosine Picked Threonine Kinase), the human homolog of MSP1 (Monopolar Spindle 1), regulates nuclear targeting of c-Abl through the 14-3-3-coupled phosphorylation site. Nihira et al. demonstrated that TTK-dependent phosphorylation of c-Abl on Y735 is required for the cytoplasmic sequestration/localization of kinase. TTK/Msp1 deficiency enhances the oxidative-stress-induced apoptosis while favoring the nuclear accumulation of c-Abl [48].

c-Abl co-localizes with the endoplasmic reticulum (ER)-associated protein grp78 [49]. Subcellular fractionation studies indicate that over 20% of c-Abl is detectable in the ER. Induction of ER stress with the calcium ionophore A23187, brefeldin A, or tunicamycin is linked to translocation of the ER-associated c-Abl fraction to mitochondria. In concert with targeting of c-Abl to mitochondria, cytochrome c is released in response to ER stress through a c-Abl-dependent mechanism. In c-Abl-deficient cells, ER-stress-induced apoptosis is attenuated thus implying the involvement of c-Abl in signaling from the ER to mitochondria [49]. Kumar et al. indicated that in response to oxidative stress, cytoplasmic c-Abl moves to mitochondria, where it mediates mitochondrial dysfunction and cell death. Moreover, targeting of c-Abl to mitochondria is also dependent on activation of PKC*δ* and relies on c-Abl catalytic activity. In the response to hydrogen peroxide, pharmacological inhibition of c-Abl with STI571 decreases c-Abl targeting to mitochondria and attenuates mitochondrial dysfunction and cell death [50, 51]. Downregulation of c-Abl or PKC*δ* impaired PARP cleavage, suggesting that both PKC*δ* and c-Abl can induce apoptosis through the mitochondrial pathway in the absence of p53, p73, and FAS upon genotoxic stress [52]. 

Taken together all these observations suggest that c-Abl activation can exert a positive role both in the intrinsic and extrinsic apoptotic signaling pathways.

## 5. Perspectives

Signaling networks are composed of multiple layers of interacting proteins. Activation of most cell signaling circuits is modulated by feedback control, and disease conditions are often caused by the loss of the feedback control. A comprehensive understanding of the complexities of the signaling network is required to design therapies that are effective without inducing off-target consequences [53]. In neurodegenerative disorders, the duration and the spatial organization of signaling complexes can cause a system failure ending in neuronal loss. Evidence compiled above indicates that c-Abl activation could act as an arbiter of neuronal cell fate under various stress conditions. Subcellular localization of c-Abl can play an important role to modulate activation and assembly of signaling networks. Pharmacological inhibition of the catalytic activity can prevent targeting of c-Abl to mitochondria and the consequent programmed cell death. In the nucleus, c-Abl signaling modulates oxidative-stress-induced transcription resulting in neuronal death. In this scenario, a new therapeutic strategy for degenerative neurological diseases may be based on the possibility to rewire the network characterizing the pathological states, by restoring a feedback control *via* inhibition of c-Abl signaling. Several types of inhibitors have been designed to target with high selectivity the c-Abl kinase by different mechanisms [54, 55]. Allosteric inhibitors repress the catalytic activity by binding to a site far from the kinase-active site. Allosteric binding does not prevent the binding of ATP-competitive inhibitors such as STI571. Experimental data provide evidence that both types of inhibitors can work in synergy to inhibit aberrant activation of Bcr-Abl [55, 56]. Insufficient or excessive inhibitor doses not only may be inefficacious but may also have adverse effects. In addition, targeting of c-Abl to different cellular compartments is linked to the catalytic domain conformation. A recent report indicates that binding of STI571 to the catalytic domain can restore the nuclear import of the Bcr-Abl mutant, suggesting that the auto-inhibited conformation of c-Abl is required for nuclear translocation [57]. Interestingly, an allosteric inhibitor, GNF-2, induces a translocation of myristoylated c-Abl to the endoplasmic reticulum, competing with the intramolecular engagement of the NH2-terminal myristate for binding to the c-Abl kinase myristate-binding pocket [58]. A priority is now the identification of effective combination therapies for native conformations of c-Abl kinases, allowing the reactivation of appropriate regulation circuits in aged neurons. As mentioned, administration of reactive oxygen species scavengers prevents the accumulation of c-Abl and p53 leading to a decreased apoptosis of NPCs. In line with this, treatment with curcumin, an activator of the antioxidant Nfr2 [59] pathway can ameliorate the neurological symptoms and survival of Niemann-Pick type C mice [60]. This suggests the possibility to develop combined targeted therapies of antioxidants in tandem with c-Abl kinase inhibitors [42]. Despite the technical hurdles, rewiring of cell signaling networks *via* inhibition of a single node, such as c-Abl, may prove an effective therapeutic strategy. 

## Abbreviations

c-Abl: Abelson tyrosine kinase
ROS: Reactive oxygen species
GSH: Glutathione
GSSH: Glutathione disulphide
SOD: Superoxide dismutases
PRxs: Peroxiredoxins
GPx: Peroxidases
DDR: DNA damage response
ATM: Ataxia telangiectasia mutated
DNAPKcs: DNA-dependent protein kinase catalytic subunit
ATR: Rad-3 related
Arg: c-Abl-related gene
GVD: Granulovacuolar degeneration
AD: Alzheimer disease
NTFs: Neurofibrillary tangles
A*β*: Amyloid-*β* fibrils
STI571: Imatinib mesylate
NPC: Niemann-Pick type C
NAC: N-acetylcysteine
NPCs: E12.5 cortical neural progenitor cells
Cdk5: Cyclin-dependent kinase 5
PKC*δ*: Protein Kinase C-delta
PD: Parkinson's disease
MPTP: 1-methyl-4-phenyl-1,2,3,6-tetrahydropyridine
MST1: Mammalian Ste20-like kinase1
FOXO3: Forkhead box O3
TTK/PYT/Msp1: (Phosphotyrosine-Picked Threonine Kinase), the human homolog of MSP1 (Monopolar Spindle 1)
ER: Endoplasmic reticulum
PARP: Poly (ADP-ribose) polymerase
Bcr-Abl: Oncogenic fusion protein (breakpoint cluster region).
